# SynthEx: a synthetic-normal-based DNA sequencing tool for copy number alteration detection and tumor heterogeneity profiling

**DOI:** 10.1186/s13059-017-1193-3

**Published:** 2017-04-08

**Authors:** Grace O. Silva, Marni B. Siegel, Lisle E. Mose, Joel S. Parker, Wei Sun, Charles M. Perou, Mengjie Chen

**Affiliations:** 1 Department of Genetics, University of North Carolina at Chapel Hill, Chapel Hill, NC USA; 2 Curriculum in Bioinformatics and Computational Biology, University of North Carolina, Chapel Hill, NC 27599 USA; 3 Lineberger Comprehensive Cancer Center, University of North Carolina, Chapel Hill, NC 27599 USA; 4 Section of Genetic Medicine, Department of Medicine, The University of Chicago, 900 East 57th Street, KCBD 3220A, Chicago, IL 60637 USA; 5 Public Health Division, Fred Hutchison Cancer Research Center, Seattle, WA 98109 USA

**Keywords:** Breast cancer, Copy number, Whole exome, Sequencing, Whole genome, The Cancer Genome Atlas, Synthetic normal

## Abstract

Changes in the quantity of genetic material, known as somatic copy number alterations (CNAs), can drive tumorigenesis. Many methods exist for assessing CNAs using microarrays, but considerable technical issues limit current CNA calling based upon DNA sequencing. We present SynthEx, a novel tool for detecting CNAs from whole exome and genome sequencing. SynthEx utilizes a “synthetic-normal” strategy to overcome technical and financial issues. In terms of accuracy and precision, SynthEx is highly comparable to array-based methods and outperforms sequencing-based CNA detection tools. SynthEx robustly identifies CNAs using sequencing data without the additional costs associated with matched normal specimens.

**Electronic supplementary material:**

The online version of this article (doi:10.1186/s13059-017-1193-3) contains supplementary material, which is available to authorized users.

## Background

Drivers of tumor growth, progression, and metastasis are often a result of alterations in gene dosage and/or structure due to copy number alterations (CNAs). In breast cancer, common disruptions of specific genomic areas are known to drive oncogenic alterations [1]. Previous research has identified key drivers in a subtype-specific manner that are a direct result of CNAs rather than somatic point mutations. These acquired genomic alterations can foster the activation of oncogenes or inactivation of tumor suppressors in cancer cells [2]. CNA detection has also previously identified therapeutic targets across multiple cancer types [3–6]. The clinical importance of accurately measuring CNAs is critical to understanding the biologic progression of cancer.

Previous efforts to identify CNAs in tumors utilized microarray-based technologies, such as array comparative genomic hybridization (aCGH) and single nucleotide polymorphism (SNP) genotyping arrays. Currently, next-generation sequencing approaches enable a comprehensive survey of all genomic variations in one sample. Furthermore, whole exome sequencing (WES) is a popular tool for cancer genomics projects as it involves a reduction in analytical complexity and financial burden compared to whole genome sequencing (WGS). With efforts from large sequencing consortia, such as The Cancer Genome Atlas (TCGA) project [7], WES data for thousands of tumors spanning a multitude of cancer types are currently available. Harnessing these technologies to accurately identify CNAs in tumor samples provides a powerful opportunity for additional research using these data.

Significant technical challenges in the detection of CNAs from sequencing platforms currently limit the use of WES data for accurate DNA copy number characterization. Errors in the human reference genome, repetitive sequences, polymorphism, and procedural bias during next-generation sequencing currently complicate copy number calling [8]. For WES data specifically, accurate copy number segmentation is further complicated by non-uniform capture efficiency of exons between two samples. Two generalized approaches to detect CNAs from WES include: reliance on depth of coverage from target regions, thus ignoring a large portion of the genome [9–13]; and utilizing uniformly distributed off-target reads [14], thus ignoring the signal necessary for sophisticated analyses such as estimation of integer copy number, sample purity, and clonality. To address these issues, we developed a method that leverages information from both off-target and on-target regions.

Previously published algorithms have attempted to address the challenges of detecting CNAs from WES; however, to our current knowledge none has provided a comprehensive solution with the additional ability to reduce the current high cost of requiring matched normals. We developed SynthEx, a tool that caters to the varying protocols of different next-generation sequencing protocols, to detect CNAs. SynthEx uses a “synthetic-normal” strategy to correct for sample-specific bias in target regions due to pre-analytical variation between tumor–normal matched pairs. Therefore, instead of requiring a matched tumor–normal paired sample from each subject, a synthetic normal is used that mimics the technical bias of the tumor to be assayed. Using published CNAs by TCGA from Affymetrix SNP 6.0 as the “gold standard”, we compared the performance of SynthEx against popular WES CNA detection methods [9, 11, 15], using TCGA breast carcinoma as the training set and TCGA head and neck carcinoma as the test set. Here, we provide a novel copy number calling tool utilizing WES data with improved precision and accuracy that does not require matched normal specimens.

## Results

### Sample-specific bias of read ratios in exonic/target regions due to fold enrichment differences

To explore new methods for assessing copy number alterations (CNAs) using short read DNA sequencing data, we utilized whole exome sequencing data from 989 TCGA breast tumors and matched normal specimens (Additional file 1) [16].

One significant challenge in calling CNAs from whole exome and targeted sequencing is how to use the information and accurately predict copy number from off-target regions. We first explored the differences in non-overlapping bin sizes in order to have >50× coverage in each bin (Additional file 2: Figure S1). Utilizing 100-kb non-overlapping bins to maximize the coverage in our exploratory analysis, we first examined whether the ratios of matched tumor-to-normal, or the read ratios (RRs), from target regions had a similar distribution in the on-target and off-target regions. If this was true, then one could directly apply existing change point methods developed for SNP array data to non-overlapping bins.

**Fig. 1:**
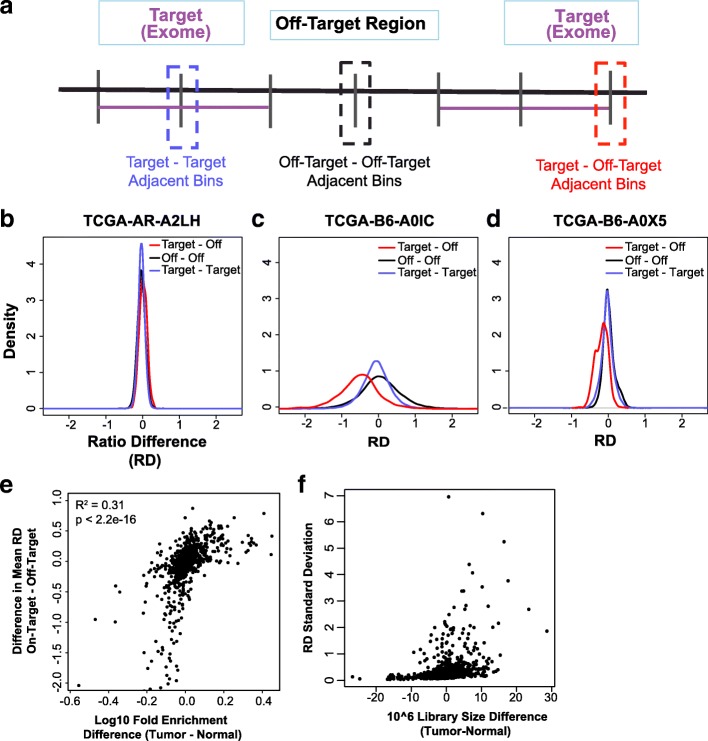
Sample-specific bias of read ratios due to sequencing quality metrics of the tumor–normal pair. **a** The three types of adjacent bins. Differences in ratio density (RD) of adjacent bins spanning on-target regions, off-target regions, or both demonstrating **b** similar distributions, **c** dissimilar distributions not centered at 0, or **d** dissimilar distributions centered on 0. **e** Mean RD compared to the difference of fold enrichment (log10 scale) between the tumor–normal pairs for 989 TCGA breast cancer samples. **f** Standard deviation of the RD compared to the difference in the library size (×10^6^) for 989 TCGA breast cancer samples

**Fig. 2:**
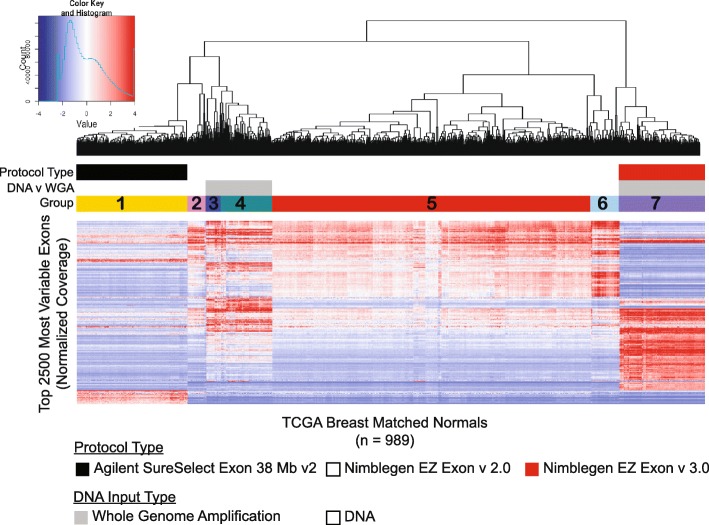
Hierarchical clustering of the top 2500 most variable exons in TCGA breast normal. Unsupervised hierarchical clustering of mean-centered coverage of the 2500 exons with the highest variability across the 989 TCGA normals. *Color bars* indicate protocol differences (*black* = Agilent, *white* = Nimblegen v 2.0, *red* = Nimblegen v 3.0) and analyte type (*gray* = whole genome amplification, *white* = DNA). Groups defined by hclust are identified by color in the bottom row

We calculated the RRs for each matched tumor–normal pair at each 100-kb non-overlapping bin. For each pair of adjacent bins, we calculated the difference of the read ratios (RDs; an abbreviation for ratio difference). Each pair of adjacent bins was then categorized into three categories based on the exon target regions: adjacent on-target bins, adjacent off-target bins, or an off-target bin adjacent to an on-target bin (Fig. 1a). Mapping the distribution of RDs from these three categories resulted in three different patterns (Fig. 1b–d): 1) RDs in the adjacent bins followed the same distribution, resulting in the density curves of the RD centralizing at 0 and having the same shapes (Fig. 1b); 2) the three bin categories followed different distributions, causing the density curves of the RDs to not centralize at 0 (Fig. 1c); or 3) the three bin categories followed different distributions, causing the density curves of the RDs to centralize on 0 but not share the same shape (Fig. 1d). The varying behavior in the density of RD from TCGA samples indicates that the RR of matched tumor–normal from target regions cannot directly compare with that from non-target regions.

To interrogate the cause of this variation, we first examined the GC content of the predicted copy number neutral bins. We observed a largely uniform distribution with minimal non-uniformity at the tail (GC >0.5). This non-linear pattern and inconsistency indicates patterns that may be due to other non-GC factors. Next, we performed single variable regression analysis of the difference in matched tumor–normal for 16 quality metrics from Picard to identify the variance of the RD as a function of these different metrics (Additional file 2: Figure S2; Additional file 3: Table S2). The coefficients of determination varied from 0.001 (GC dropout) to 0.31 (fold enrichment) (Additional file 2: Figure S3a). Interestingly, fold enrichment was also highly correlated with roughly half of the other Picard metrics (Additional file 2: Figure S3b). Here, fold enrichment is defined as the amount of fold change in which the target region is amplified above genomic background. As fold enrichment during whole exome capture differs between the tumor and matched normal, the distribution of RD in the target bins shifted with respect to their adjacent off-target bins (Fig. 1e).

A second important variable in altering the RD was the differences in library size. When the library sizes of the tumor and matched normal differed significantly, a greater standard deviation was observed (Fig. 1f). Taken together, library size and fold enrichment are two significant factors contributing to the technical bias introduced when comparing tumor to matched normal for quantifiable copy number calling.

### Creating a synthetic normal library

In order to address the technical bias created by differences in library size and capture efficiency in whole exome DNA sequencing, we utilized a synthetic normal strategy to replace matched normal. We began by performing unsupervised hierarchical clustering of the mean-centered coverage of the top 2500 exons with the largest variance across the 989 TCGA breast normal set (Fig. 2). Normal samples are assumed to be diploid, and thus we hypothesized that any resulting structure is indicative to technical biases. Seven distinct patterns of coverage across these exons were noted in the unsupervised clustering (i.e., not supervised by knowledge of protocol differences). We then investigated whether these were differences due to processing features. Protocol versions of Nimblegen v 2.0, Nimblegen v 3.0, and Agilent SureSelect technologies were specifically associated with different clusters, highlighting that even different versions of exon capture protocols can affect targeted capture efficiency. Additionally, the type of initial analyte used significantly grouped with specific clusters (Fig. 2a, color bar).

**Fig. 3:**
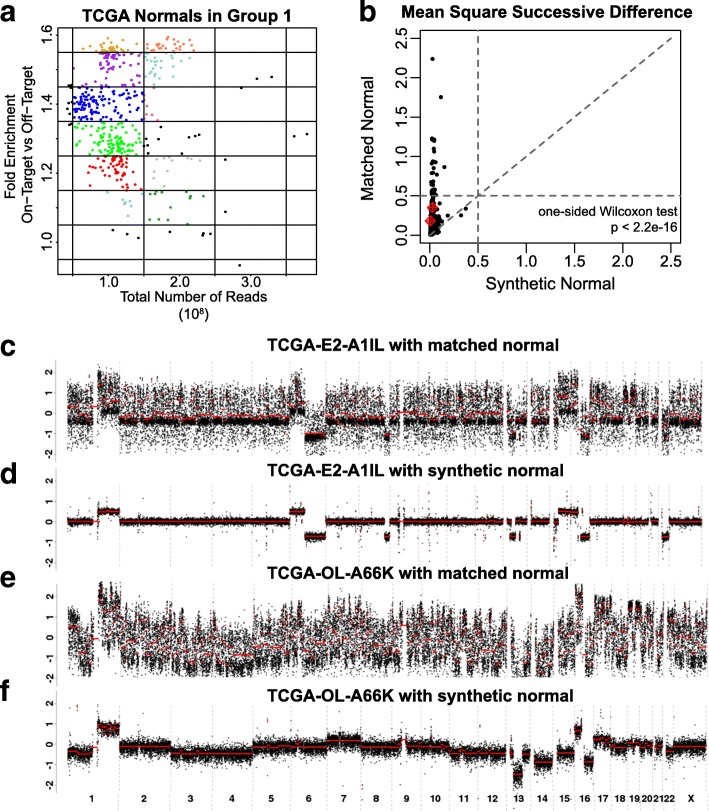
Generation of synthetic normal and improvement of technical noise with synthetic normal. **a** Library size and fold enrichment of TCGA normals. Each color represents the normal utilized in generating the synthetic normal for that bin. **b** Comparison of mean square successive difference calculated by using a matched normal (*y-axis*) or a synthetic normal (*x-axis*) for all 989 TCGA breast cancer samples, with tumors from **c**–**f** marked in *red*. Read ratios plotted by genomic location of two TCGA breast cancers and normals from the same patient (**a**, **c**) compared to read ratios calculated from the same two tumors using a synthetic normal (**b**, **d**)

We anticipated that matching tumors to normal based on library size and fold enrichment may reduce technical variation relative to the actual subject’s matched normal. Within each of the seven exome clusters identified through hierarchical clustering, we grouped normal samples by library size and fold enrichment (Fig. 3a). For each bin, we averaged the normal samples within that bin to generate a representative “synthetic” normal. To call CNAs from a tumor sample, we first calculated the fold enrichment and library size of the tumor. Then we selected the synthetic normal with similar fold enrichment and library size from each of the seven exome groups. Finally, we compared the variance of the tumor to each of the possible seven synthetic normals from the previously defined exome groups and selected the synthetic normal with the least variance. Thus, the SynthEx Synthetic method utilizes the most ideal pooled synthetic normal based on library size and fold enrichment.

In order to test for differences between subject matched and SynthEx Synthetic, we assume RR to be a piecewise constant function of genomic location and measure the magnitude of variation with the mean square successive difference (MSSD) to represent the amount of technical bias. The mean MSSD for all 989 TCGA breast cancer samples is lowered from 0.09 to 0.02 when using a synthetic normal versus using the matched normal sample (Fig. 3b; one-sided Wilcoxon test, *p* value <2.2e-16). Specifically, 90% of the tumor samples (*n* = 896) have an improved MSSD value. Furthermore, there’s a striking difference and reduction of noise in the RR plots of two tumors analyzed using a matched normal (Fig. 3c, e) versus using a synthetic normal (Fig. 3d, f).

### Varying bin sizes

To assess the robustness of SynthEx Synthetic to bin sizes at varying library sizes (8–30 million reads), we evaluated the performance of our method at 10-, 20-, 50-, and 100-kb non-overlapping bins. We first calculated the percentage of tumors which had at least 20 reads in 90% of the bins (Additional file 2: Figure S1). At 10-kb resolution, 49% of samples have at least 20 reads in ≥90% quantified bins. At 20-kb resolution, 78% of samples have sufficient coverage in 90% quantified bins. At 50 kb, the coverage plateaus with 93% of samples having adequate coverage. Furthermore, the MSSD at each overlapping bin size significantly decreases with increasing bin size (Additional file 2: Figure S4a; ANOVA *p* value <2e-16). This suggests that 10- and 20-kb bin sizes may not adequately span the genomic space to accurately call CNAs.

We next tested our original assumption that 100-kb bin sizes, which provided the highest coverage of the genome with 50× coverage per bin, was an improvement over 10-, 20-, or 50-kb bins. We calculated Jaccard Index (JI), sensitivity, and specificity relative to Array SNP CNA segments as statistical evaluations of the precision and accuracy of our tool (Additional file 2: Figure S4b–d). ANOVAs demonstrate no significant difference for all three statistical measures across the various bin sizes, although there is a trend of a slight trade-off of sensitivity and specificity when comparing 10 to 20 kb and 50 to 100 kb (ANOVA *p* value: JI = 0.63; sensitivity = 0.96; specificity = 0.871).

### Alternative approaches using the synthetic normal strategy

We investigated utilizing the K nearest neighbor (KNN) strategy as an alternative approach to generating a new synthetic normal without using fold enrichment information. Given a tumor sample, we scan through all of the available normal samples, calculate the MSSD for each normal, and select “K” normals with the smallest variance MSSD value. SynthEx then generates the new synthetic normal by taking the median across the K selected normals. This generated synthetic normal is then used with the tumor sample for copy number determination. We call this the K nearest neighbor (KNN) strategy. Compared to using SynthEx Synthetic with the large library of synthetic normals, this approach is more appropriate for studies with few normal samples or for a facility where the protocol is constantly changing.

**Fig. 4:**
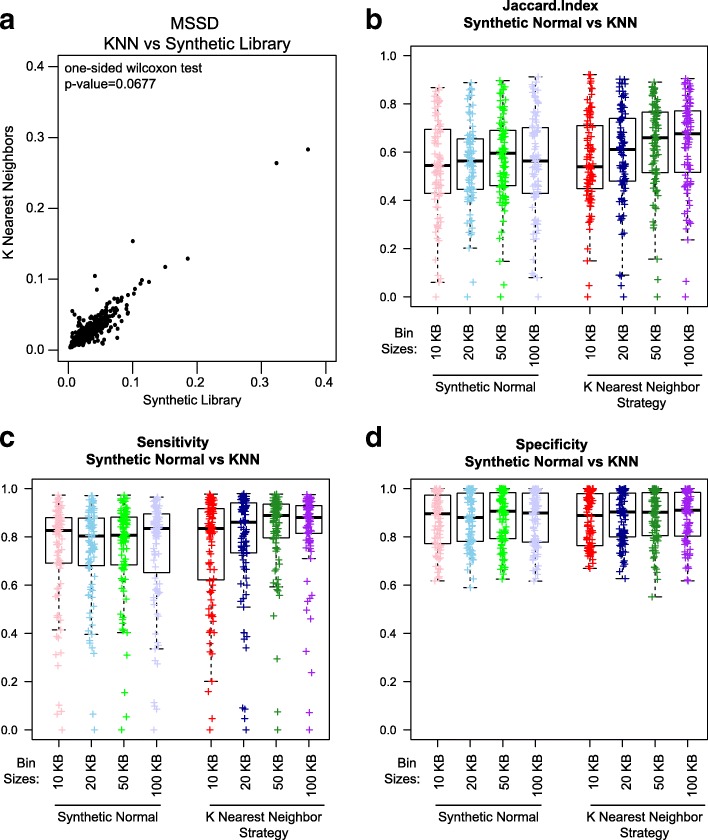
Varying bin sizes for the synthetic normal and K nearest neighbor. Copy number ratios were calculated from the K nearest neighbor strategy for K = 5 at varying bins and compared to SynthEx Synthetic (composite synthetic normal) using: **a** the mean square successive difference at 100-kb non-overlapping bin sizes; **b** the Jaccard Index; **c** sensitivity; and **d** specificity using SNP array as the ground truth at 10 (*red*), 20 (*blue*), 50 (*green*), and 100 kb (*purple*)

For TCGA BRCA samples (989), copy number for each tumor was re-calculated using a synthetic normal generated from K = 5 normals. Variance was calculated by MSSD, and these values were compared to MSSD when using a library of pre-defined synthetic normal as described above. MSSD values were highly correlated (Fig. 4a; R^2^ = 0.924; one-sided Wilcoxon test, *p* value = 0.0677). This indicates KNN can be used as a generalization of the cluster-based solution.

We again explored whether 100 kb was the appropriate bin size. Using 10-, 20-, 50-, and 100-kb non-overlapping windows with K = 5, we tested the KNN method compared to the synthetic normal strategy, calculating JI, sensitivity, and specificity compared to Array SNP CNA as the gold standard (Fig. 4b–d). There is a significant improvement of the JI with increasing bin size (ANOVA of KNN bins *p* value = 0.021, F = 3.272) as well as a slight trade-off of bin size for sensitivity (ANOVA of KNN bins *p* value = 0.023) but not specificity (ANOVA of KNN bins *p* value = 0.855).

### Concordant copy number calling with SynthEx

To evaluate the performance of our synthetic normal strategy relative to previously published methods, we compared a subset of TCGA breast tumor CNAs assayed by three platforms: Affy SNP 6.0 (SNP), whole exome sequencing (WES), and whole genome sequencing (WGS) platforms (“BRCA”, *n* = 92; Additional file 1: Table S1) [17]. BRCA WGS SynthEx CNA landscape plots more closely resemble SNP CNA landscape plots than plots created using the TCGA WGS CNAs from tumor–normal matched pairs (Additional file 2: Figure S5). Furthermore, less noise is observed in the WGS CNA landscape plot from SynthEx compared to the TCGA WGS CNAs landscape plot (Additional file 2: Figure S5b,c).

**Fig. 5:**
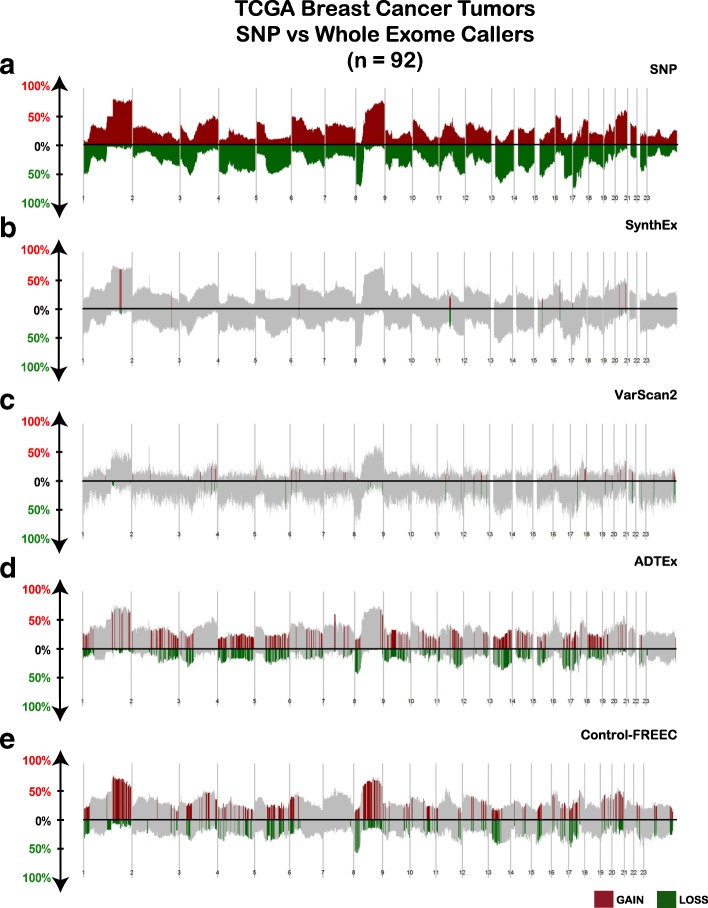
CNA genomic landscape comparing SNP arrays and whole exome sequencing tools in TCGA breast cancers. Using SWITCHplus, segments of copy number gained (above *x-axis*) and lost (below *x-axis*) are plotted from **a** SNP array, **b** SynthEx, **c** VarScan2, **d** ADTEx, and **e** control-FREEC. The frequency of an alteration out of the 92 tumors analyzed in each tool is indicated on the *y-axis* from 0–100%. Regions in *red* (gains) or *green* (loss) indicate segments that contain at least one gene whose discrete copy number alteration direction call is significantly different from SNP by Wilcoxon test

The same 92 BRCA patient WES data were analyzed with VarScan2 [15], ADTEx [9], and Control-FREEC (Table 1, Fig. 5) CNA detection tools. CNAs determined from Affy SNP data are used as the gold standard to which all WES CNA detection tools are compared. Genome-wide CNA frequency landscape plots from SynthEx, VarScan2, ADTEx, and Control-FREEC produced similar plots as the SNP-based copy number landscape plot (Fig. 5). Furthermore, expected regions of frequently occurring copy number gains at 1q and 8q and copy number losses at 1p, 5q, and 8p were also identified across all landscapes plots from both WES and WGS data (Fig. 5; Additional file 2: Figure S5).

**Table 1:** Comparison of somatic copy number detection tools from whole exome sequencing data

	ADTEX	Control-FREEC	SynthEx	VarScan2
Programming language	R, Python	C++	R, Python	Java, Perl, R
Sequencing type	WES	WES/WGS	WES/WGS	WES
Input files	BAM, coverage	SAM/BAM, pileup	BAM	BAM, pileup
Matched normal required	Yes	No	No	Yes
Window/binning	Exons	Non-overlapping windows in exons	Non-overlapping windows of genome	Non-overlapping windows in exons
Bias correction	No	Optional	NA	No
Purity estimate	No	Optional	YES with variant calling	No
Segmentation method	CBS	Lasso	CBS	CBS
Segmentation annotation	Yes	Yes	Yes	Yes
Graphics provided	Yes	Yes	Yes	Yes

*CBS* circular binary segmentation

**Fig. 6:**
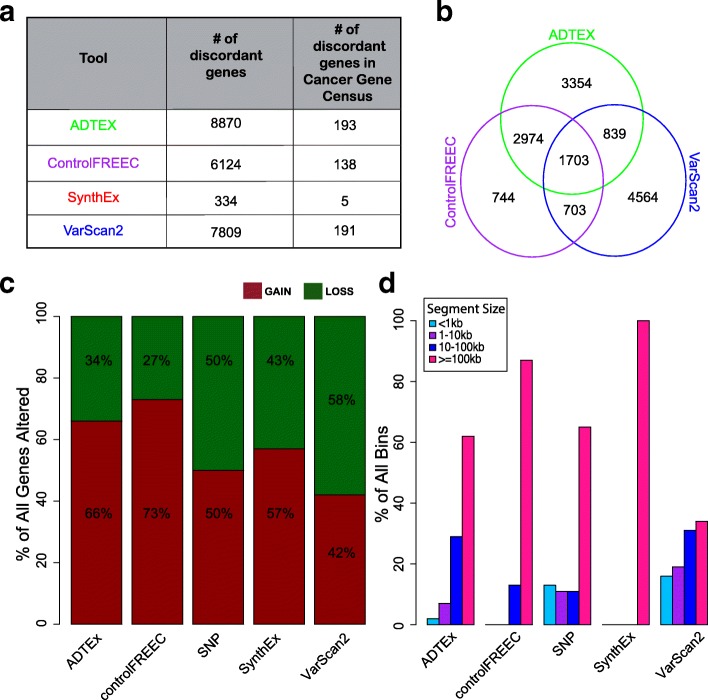
Quantification of differences in CNA calls from the WES CNA detection tools. For each tool, **a** the number of genes significantly different compared to SNP arrays and **b** the overlap of those genes across platforms. **c** Proportion of all calls that are copy number gains and copy number loss segments called by each WES CNA detection tool. **d** Lengths of copy number segments detected by each WES CNA detection tool compared to the SynthEx synthetic normal strategy using 100-kb non-overlapping bins

Genes whose discrete copy number calls differed significantly from the discrete SNP gene-level copy number by Wilcoxon tests are highlighted (Fig. 5) and quantified (Fig. 6a, b). Discrete gene-level copy number calls using SynthEx shared a 98% overlap with SNP array-based calls, with 334 genes discordant between SNP and SynthEx and five of these genes annotated within the Cancer Gene Census [18]. ControlFREEC (69%), VarScan2 (60%), and ADTEx (55%) shared less overlap with the gene-level CNAs from SNP arrays. Of the genes discordantly called in each software, 1703 were consistently miscalled among ADTEx, VarScan2, and ControlFREEC (Fig. 6b). Furthermore, noticeable variations in the percentage of observed copy number gains versus copy number losses occurred across the different WES methods, with SynthEx calls most closely resembling the fraction of CNAs produced by SNP (Fig. 6c). Finally, each method produced varying copy number segment sizes, with SynthEx having a fixed bin size of 100 kb (Fig. 6d).

### Assessing precision and accuracy of segments of SynthEx

**Fig. 7:**
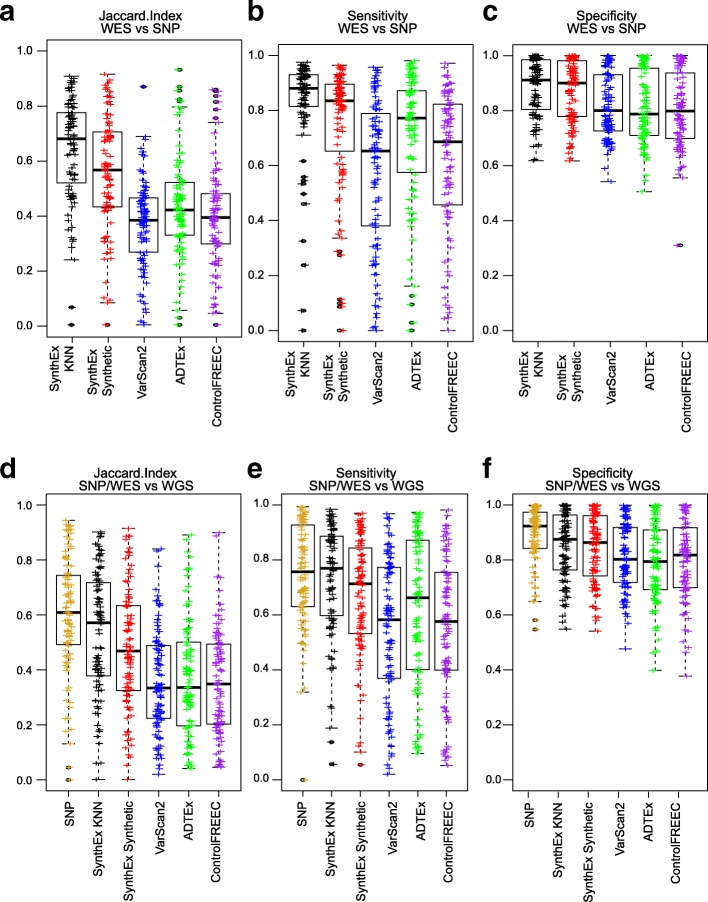
Jaccard Index, sensitivity, and specificity of SynthEx. Statistics of CNA tools from whole exome sequencing (*WES*) methods SynthEx KNN (*black*), SynthEx Synthetic (SN, *red*), VarScan2 (*blue*), ADTEx (*green*), and ControlFREEC (*purple*) compared to SNP arrays (**a**–**c**) and whole genome sequencing (*WGS*) (**d**–**f**) in TCGA breast cancer dataset. Compared to SNP arrays, one-sided *t*-tests tested SynthEx SN to the next highest mean to determine significance for **a** Jaccard Index (SynthEx SN versus ADTEx *p* = 4.9e-5), **b** sensitivity (SynthEx SN versus ADTEx *p* = 0.40), and **c** specificity (SynthEx SN versus VarScan2 *p* = 5.6e-10). Overall mean differences were significant in all plots as measured by ANOVA (Jaccard Index *p* = 1.7e-8, sensitivity *p* = 0.00029, specificity *p* = 0.0055). Compared to WGS, SNP (*gold*) and SynthEx KNN and SynthEx SN outperformed the other WES callers: **d** Jaccard Index (ANOVA *p* < 2e-16), **e** sensitivity (ANOVA *p* = 5.4e-9), and **f** specificity (ANOVA *p* = 1.5e-8). No statistical differences between SNP and SynthEx SN were measured in **d**–**f** (one-sided *t*-test *p* > 0.94)

To quantify the precision and accuracy of SynthEx compared to the other WES CNA detection tools, the JI, sensitivity, and specificity were calculated at 100-kb non-overlapping bins (Additional file 4: Table S3). In BRCA, comparing each method to the SNP CNAs, the median JI values are highest for SynthEx KNN (0.622) and SynthEx Synthetic (SynthEx SN; 0.526), with ADTEx following (0.418) (Fig. 7a, one-sided t-test ADTEx v. SynthEx SNp = 4.9e-5; ANOVA p = 1.7e-8). VarScan2 and ControlFREEC have lower JI values (VarScan2, 0.380; ControlFREEC, 0.390). SynthEx also outperformed the other copy number detection WES tools within the BRCA genomically defined and clinically heterogeneous breast cancer subtypes (Additional file 2: Figure S6), which have known variations in CNAs and genomic drivers [19, 20].

The median sensitivity of the WES CNA detection tools compared to SNP-based CNAs demonstrates a modest improvement using SynthEx KNN (0.88), SynthEx Synthetic (0.84), and ADTEx (0.77; one sided *t*-test *p* = 0.40) but a significant improvement compared to Control-FREEC (0.69; *p* = 2.5e-4) and VarScan2 (0.65; *p* = 2.4e-7) (Fig. 7b; overall ANOVA *p* = 0.00029). The median specificity follows a similar pattern, with SynthEx KNN and SynthEx Synthetic outperforming the other callers (Fig. 7c; SynthEX KNN, 0.91; SynthEx Synthetic, 0.90; VarScan2, 0.80; Control-FREEC, 0.80; ADTEx, 0.79; one-sided *t*-test *p* < 2.5e-10; ANOVA *p* = 0.0055). In addition, SynthEx Synthetic continued to outperform the other WES detection tools in terms of median sensitivity and specificity when the comparison was subdivided into the intrinsic breast cancer molecular subtypes (Additional file 2: Figures S7 and S8). Finally, for all bin sizes, both the KNN and Synthetic strategies outperform previously published WES detection tools ADTEx, VarSacn2, and Control-FREEC (Additional file 2: Figure S9).

To further assess the robustness of SynthEx, we compared the WES tools and SNP arrays using the TCGA WGS CNAs as the gold standard (Additional file 5: Table S4). For each measurement of precision or accuracy, SNP and SynthEx Synthetic were not significantly different from one another (all one-sided *t*-tests SNP versus SynthEx >0.94). For the median JI, SNP (0.61), Synthex KNN (0.57), and SynthEx Synthetic (0.47) outperform the other WES copy number tools (Figure 7d; Control-FREEC, 0.35; ADTEx, 0.34; VarScan2, 0.33; ANOVA *p* < 2e-16). SynthEx KNN (0.77), SynthEx Synthetic (0.71), and SNP (0.76) outperform all other methods in terms of median sensitivity (Fig. 7e; ADTEx, 0.66; Control-FREEC, 0.58; VarScan2, 0.58; ANOVA *p* = 5.4e-9). Finally, all CNA detection tools have extremely high median specificity compared to WGS CNAs, with SynthEx and SNP significantly different to the other methods (Fig. 7f; SNP, 0.92; SynthEx KNN, 0.88; SynthEx Synthetic, 0.86; Control-FREEC, 0.82; VarScan2, 0.80; ADTEx, 0.79; ANOVA *p* = 1.5e-8).

Assuming that the normals collected in TCGA breast dataset were diploid, we tested the false positive rate of the SynthEx Synthetic caller. SynthEx Synthetic at 100-kB bin size called a median of 0.0083 bins in the human genome as altered (0.0080–0.02 bins).

### Validation of SynthEx in TCGA head and neck squamous cancers

To validate the findings seen for the BRCA dataset, we repeated the above analyses using a subset of TCGA head and neck squamous cellular carcinomas with both SNP and WES platforms (“HNSC”; *n* = 100; Additional file 6: Table S5). SynthEx HNSC CNAs most closely match the SNP copy number landscape plots compared to plots generated from VarScan2, ADTEx, and Control-FREEC (Additional file 2: Figure S10). Previously published highly frequent copy number gains at 3q, 5p, and 8q and copy number losses at 3p and 8p were observed in the HNSC copy number landscape plots. Discrete gene-level copy number calls using SynthEx shared a 97% overlap with SNP-based gene level copy number calls within HNSC (Additional file 2: Figure S10).

Examining the variation in bin size for HNSC, we next compared the KNN and synthetic normal strategies. No significant differences were observed for JI, sensitivity, or specificity at varying bin sizes or with the different strategies (Additional file 2: Figure S11a-c).

Examining the same statistical metrics using SNP arrays as the gold-standard, SynthEx’s performance was competitive with other WES CNA tools (Additional file 7: Table S6) that require matched normals. The median JI was higher in SynthEx KNN (0.71) and SynthEx Synthetic (0.67) compared to the other WES CNA detection methods (Additional file 2: Figure S11d; ANOVA *p* < 2e-16; one-sided *t*-test versus VarScan2 *p* = 4e-14). Although the median sensitivity of VarScan2 (0.94) and ADTEx (0.97) slightly out-performed SynthEx KNN (0.93) and Synthetic (0.93) in the HSNC cohort, all three values are extremely high and not statistically different (Additional file 2: Figure S11e; one-sided *t*-tests: SynthEx Synthetic versus VarScan2 *p* = 0.99; SynthEx Synthetic versus ADTEx *p* = 0.99; SynthEx Synthetic versus ControlFREEC p = 6.8e-16). In addition, SynthEx KNN (0.94) and Synthetic (0.95) outperformed the other WES copy number tools in terms of specificity (Additional file 2: Figure S11f; ANOVA *p* = 6.6e-11; one-sided *t*-tests, SynthEx versus VarScan2 *p* = 1.1e-15, SynthEx versus ADTEx *p* < 2.2e-16, SynthEx versus ControlFREEC *p* < 2.2e-16). Cumulatively, SynthEx outperforms the other WES CNA detection tools in a reproducible manner.

## Discussion and conclusions

We present SynthEx, a novel tool that detects CNAs from both whole genome and whole exome sequencing (WES) data by comparison to a synthetic normal. We strongly emphasize the differences in exon capture patterns observed both across and within protocols, which must be considered when utilizing sequencing data for CNA calling. We demonstrate that SynthEx outperforms ADTEx, Control-FREEC, and VarScan2 CNA detection tools in both accuracy and precision.

Our experience suggests that technical variation in WES goes beyond differences in experimental protocols. Utilizing read ratios (RRs) to call copy number alterations has an underlying assumption that generation of the on-target and off-target regions have non-significant variation in the tumor and matched normal. Here, we demonstrate that accounting for this technical noise is critical to accurately determine CNAs from next-generation sequencing technologies. SynthEx provides a robust method to handle technical variation and a collection of heterogeneous normal samples. To the best of our knowledge, SynthEx is the first tool that utilizes a matching synthetic normal based on the consistency of target sequencing profiles to detect sequence-based CNAs.

Many advantages for cancer researchers may exist when utilizing SynthEx over other WES CNA detection methods. The robust synthetic normal strategy does not require a matching normal sample for each tumor sample, thereby potentially reducing sequencing costs by half. Additionally, SynthEx uses both on-target and off-target reads and is thus able to accurately determine copy number across the entire genome. Finally, the use of a synthetic normal with similar quality metrics as the tumor being interrogated leads to superior performance compared to using the matched normal for both WES and WGS copy number data.

SynthEx requires some normals to be sequenced with the same protocol and/or generated by the same sequencing facility as the tumors to which they are compared. If a large pool of samples is available (i.e., in large consortia like TCGA), then the pooled synthetic normal strategy can be employed. For smaller datasets, the KNN strategy can be used. Given a tumor sample, SynthEx will select the best normals that minimize the technical variability of a pair. The performance of SynthEx is not guaranteed if using normals generated by different protocols.

In this post-data collection era of TCGA project, we foresee that many studies will integrate multiple TCGA DNA sequencing samples that are processed by different protocols. Additionally, we foresee that large consortia will encounter similar issues with changes in protocols over time. Thus, it will be necessary to closely examine technical artifacts when performing any quantitative analysis, especially in the context of copy number detection. Recognizing that CNAs are essential steps in tumorigenesis and metastasis in many tumor types, it is critical to robustly and accurately determine CNAs from sequencing data. SynthEx offers a unique solution for analysis of heterogeneous samples from large genomics projects in this regard.

Due to the inherently heterogeneous, interrupted coverage of the genome by targeted/whole exome sequencing, sequencing reads are not evenly distributed across the genome. To utilize information from off-target regions, SynthEx uses a generous fixed bin size (10–100 kb) to make sure each bin has adequate coverage (for samples with 8–30 million reads). Lower resolutions could provide CNAs within a single gene, though there is an increasing amount of noise. Even at a resolution of 10 kb, SynthEx outperforms alternative methods compared with SNP array-based calls at both the base-pair and discrete gene level.

A significant limitation of SynthEx is the inability to identify focal changes or aberrations that span only several hundred base pairs. This could be alleviated by introducing adaptive bin sizes. Several algorithms have been developed to accommodate the non-uniformity of read distribution. For example, Zhao et al. [21] proposed a “restriction-imposed” flexible binning algorithm, which generated bin sizes locally to ensure even variance as well as adequate number of reads per window. A similar algorithm has been applied in Ginkgo [22], a recent copy number calling method for single-cell sequencing data. Extending our framework to generate adaptive bin sizes and assessing the potential benefits is a promising avenue for future research.

Compared to conventional copy number analysis, which usually estimates the total copy number for a given genomic window, allele-specific copy number analysis (ASCN) is becoming increasingly popular due to its promise in clonality analysis [23–25]. ASCN methods require read counts and allele frequency at each single nucleotide as input data to infer high-resolution allele-specific copy number and accurate tumor purity/ploidy. It is worthwhile to investigate whether the synthetic normal strategy can enhance the power of ASCN methods by reducing unwanted variation at the single nucleotide level. Combining SynthEx with ASCN procedures is likely to be another fruitful future direction.

## Methods

### Breast cancer tumor datasets

For these comparative studies, two human datasets were used: the training dataset contains breast carcinoma data collected by TCGA and available via TCGA data portal (BRCA, *n* = 92) and the validation dataset contains head and neck squamous cell carcinomas collected by TCGA (HNSC, *n* = 100). The synthetic normal dataset is comprised of 989 matching normal WES samples from a larger available TCGA BRCA tumor cohort. Detailed biospecimen collection and sample processing information, including sample inclusion criteria, sample processing, and clinical data quality assessment of biomarkers, have been previously described [1, 26]. Demographic and clinical information is available (Additional file 1: Table S1).

For both BRCA and HNSC tumor samples, SNP and DNA WES data were collected from TCGA data portal. In addition, DNA WGS data were collected from a subset of matched tumor–normal BRCA samples [17]. For SNP data, the publically available level 3 circular binary-segmented copy number data were downloaded from TCGA data portal. For WES, burrows-wheeler aligner (BWA) aligned BAM files were acquired from TCGA and realigned using an assembly-based re-aligner (ABRA) with the default parameters [27, 28].The genotype-calling pipeline was built using Freebayes [29]. For WGS, BWA alignments of paired 100-nucleotide reads were acquired from TCGA and processed as previously described [16, 17].

### Calculating variability and quality assessment of sequence experiments

Using the ABRA re-aligned BAM files, we calculated read ratios (RR) in the tumor and paired normal sample for each 100-kb non-overlapping bin. Bins are classified as target bins if the bin overlaps with any selectively amplified targets. Bins that do not overlap any selective amplified targets are labeled as off-target bins. Furthermore, bins are also grouped into adjacent bins. Then, each pair of adjacent bins is divided into three categories: two-adjacent target bins, two adjacent off-target bins, or a target bin adjacent with an off-target bin. The variability is assessed by ratio difference (RD), calculated as the difference of the RR between the two bins that comprise the adjacency bins. For each sample, the distribution of RD is plotted and mean and variance are calculated.

Using Picard (http://broadinstitute.github.io/picard/), we collected various sequence-based metrics, including fragment length, sequence content, alignment, capture bias and efficiency, coverage, variant call metrics, and fold enrichment. Fold enrichment is defined as the amount of fold change in which the baited region is amplified above genomic background (http://broadinstitute.github.io/picard/). We performed regression analysis using the lm function in R v.3.3.0 on each collected metric relative to the mean of RD to determine any association between the RRs and the various quality metrics. In addition, the median minor allele frequency (MAF) is calculated for each bin. Bins with a median MAF greater than 0.45 are candidate copy neutral events. A Gaussian mixture model for RR and a Bayesian Information Criteria (BIC) are used to determine the copy number state and identify diploid bins. We assign the mixture component with the smallest mean RR as diploid. The RRs in all bins are adjusted so that the diploid regions have expected RR equal to 1. To assess the quality of the genotype calling method, we examined the exon regions covered by WES and SNP array technologies.

### Copy number calculation from synthetic normal and purity estimation

Using the BRCA WES normal samples, we created synthetic normal WES profiles that cover a spectrum of fold enrichment levels and library size levels. For each library size and fold enrichment, we averaged the normal samples that fall into that given bin. For a given tumor sample, we first analyzed the fold enrichment and library size, then searched for a matching synthetic normal. To account for samples with aneuploidy, we identified the diploid genome within the tumor sample by gauging information from allele frequencies of heterozygous sites. We used the circular binary segmentation (CBS) algorithm to identify significant change-points across RR bins and identify segment-level CNAs [30].

To accurately estimate purity, we refined a previously published WGS computational framework, SomatiCA [31]. We implemented SomatiCA’s fully specified Bayesian normal mixture model to assign each segment an integer copy number based on posterior probabilities. We further utilized several heuristic-based filters to assist the assignment of integer copy numbers which threshold the minimum number of segments in each integer copy level (gains >.25; losses <-.32 in log2 transformed ratios) and identify the minimum distance in MAF between two copy number levels.

### Selection and processing of algorithms to detect somatic copy-number alterations

We compared SynthEx against published algorithms that detect CNAs from cancer genome sequence information. Using a comparative-based literature search for top scoring CNA detection tools resulted in three algorithms: ADTEx [9], ControlFREEC [11], and Varscan2 [15]. Table 1 highlights the main features of the selected algorithms and our new tool. The dominant strategy to detect CNAs from WES data is to identify change points in the RR counts or depth of coverage ratios between a tumor and its paired normal sample at local genomic regions. ControlFREEC and VarScan2 use non-overlapping binning windows in exons to infer raw copy numbers, whereas ADTEx uses exon regions. Furthermore, ControlFREEC performs GC content normalization and mappability bias correction when inferring copy numbers. Segmentation is performed following the initial copy number identification to identify regions of the genome with shared copy number values. ADTEx and VarScan2 use CBS whereas ControlFREEC uses a lasso-based algorithm to delineate segments of similar copy number.

We applied ADTEx v.1.0.4 with default settings. We applied Control-FREEC v.7.2 with the following configuration: coefficientOfVariation = 0.05, breakPointThreshold = 0.8, window = 50000, intercept = 0, contaminationAdjustment = TRUE. VarScan2 v2.2.4 was run with mpileup default parameters: -q 1 -f ref.fa normal.bam tumor.bam | java -jar VarScan.jar copynumber varScan --mpileup 1; java -jar VarScan.jar copyCaller varScan.copynumber --output-file varScan.copynumber.called

[--output-homdel-file varScan.copynumber.called.homdel] such that the output is in log2 transformed space and comparable to SNP Array.

### Statistical analysis

To evaluate the ability of SynthEx to detect CNAs (i.e., gain or loss of genomic DNA), we compared WES and WGS SynthEx segment-level and gene-level copy number value against CNAs produced from genome-wide SNP arrays and against WES copy number detection tools, including ADTEx, ControlFREEC, and VarScan2. Discrete gene-level copy number calls are created using modification from SWITCHplus [19]. Specifically, we assigned 1 to all significant copy number gained segments identified through CBS and −1 to all significant segments of copy number loss identified through CBS; all other segments were labeled 0. Using the *copyNumberHeatmap* and *createCNGeneHeatmap* function from SWITCHplus we created copy number gene matrices for each copy number detection tool and each dataset.

Multiple statistical tests were used to assess the accuracy and precision of CNAs identified using SynthEx against other WES, WGS, and SNP-based copy number detection algorithms. Gene-level Wilcoxon tests were performed for each gene in the discrete copy number gene matrix (from WES data) against the matching gene’s discrete copy number value from the SNP (or WGS) gene matrix. Genes whose discrete call differed significantly against segments from SNP (or WGS)-based copy number profiling tools were identified as having a Wilcoxon false discovery rate *p*-value less than 0.05. Using the plotting capabilities from SWITCHplus, significantly different genes were highlighted by color on the copy number frequency landscape plot according to direction of the alteration (i.e., red for copy number gain and green for copy number loss).

Jaccard Index (JI), sensitivity, and specificity values are calculated (per sample) using segment-level CNAs from WES-based tools and SNP (or WGS) CNAs as the ground truth value, as previously described [10]. The JI calculates the amount of concordance between the genomic location of a segment’s ground truth (from SNP or WGS). Specifically, it uses set theory to represent the ratio of the intersection of two sets of genome-wide CNAs to the union of two sets of genome-wide CNAs. Sensitivity measures the length of overlapping genomic regions between a tool’s CNAs and the ground truth’s CNAs divided by the length of the ground truth’s CNAs. Specificity measures the length of non-overlapping genomic regions between the ground truth’s CNAs and the tool’s CNAs divided by the length of CNAs not called by the ground truth. Each resulting JI, sensitivity, and specificity per sample value is plotted onto a box plot and separated per copy number profiling tool. For all cohort-wide box plots, one-sided paired *t*-tests comparing the two highest means (excluding SynthEx KNN) are reported to identify whether SynthEx Synthetic Normals significantly improved the statistical measurement. ANOVA *p* values are additionally calculated and reported. For subtype-specific box plots, the ANOVA *p* value is reported to identify whether the means differed significantly among the various tools. All statistics and graphs were generated using R v.3.3.0.

## Additional files

Additional file 1: Table S1. TCGA breast cancer clinical information. (XLSX 104 kb)

Additional file 2: Figure S1. Coverage of bins at varying window sizes. **Figure S2.** Visualization of sequencing quality metrics. **Figure S3.** Condensed summary of the influence of Picard metrics on ratio differences (RD) and correlation within the Picard metrics. **Figure S4.** Statistical comparison of varying bin sizes for SynthEx. **Figure S5.** Differences in CNA landscape compared to whole genome with and without a synthetic normal. **Figure S6.** Jaccard Index of WES tools for each breast cancer subtype. **Figure S7.** Sensitivity of WES tools for each breast cancer subtype. **Figure S8.** Specificity of WES tools for each breast cancer subtype. **Figure S9.** Comparing all SynthEx strategies to other CN detection methods with TCGA BRCA. **Figure S10.** Validation of SynthEx with TCGA head and neck squamous cellular carcinoma SNP and whole exome data. **Figure S11.** Statistics of SynthEx with TCGA head and neck squamous cellular carcinoma SNP at varying bin sizes and compared to other whole exome methods. (PDF 6411 kb)

Additional file 3: Table S2. Sequencing quality metric scores collected by Picard for 989 TCGA breast cancers and matched normals as well as the mean ratio difference between matched tumor and normal. (XLSX 450 kb)

Additional file 4: Table S3. Jaccard Index, sensitivity, and specificity values in comparing SNP arrays to whole exome callers ADTEx, ControlFREEC, SynthEx, and VarScan2 for TCGA breast cancer tumors (*n* = 92). (XLSX 150 kb)

Additional file 5: Table S4. Statistical measurements comparing SNP arrays and whole exome callers to whole genome copy number data utilizing tumor and matched normal as the ground truth in TCGA breast cancer (*n* = 92). (XLSX 107 kb)

Additional file 6: Table S5. TCGA head and neck squamous cell carcinoma clinical information of tumors used in comparisons (*n* = 100). (XLSX 55 kb)

Additional file 7: Table S6. Statistical measurements comparing SNP arrays to whole exome callers for TCGA head and neck dataset. (XLSX 97 kb)
