# Supplementary material for: SynthEx: a synthetic-normal-based DNA sequencing tool for copy number alteration detection and tumor heterogeneity profiling
Source: Genome Biol. 2017 Apr 8;18:66. doi: 10.1186/s13059-017-1193-3 (PMC5385048; doi:10.1186/s13059-017-1193-3)
Supplement: Supplementary file 2 — Coverage of bins at varying window sizes. Figure S2. Visualization of sequencing quality metrics. Figure S3. Condensed summary of the influence of Picard metrics on ratio differences (RD) and correlation within the Picard metrics. Figure S4. Statistical comparison of varying bin sizes for SynthEx. Figure S5. Differences in CNA landscape compared to whole genome with and without a synthetic normal. Figure S6. Jaccard Index of WES tools for each breast cancer subtype. Figure S7. Sensitivity of WES tools for each breast cancer subtype. Figure S8. Specificity of WES tools for each breast cancer subtype. Figure S9. Comparing all SynthEx strategies to other CN detection methods with TCGA BRCA. Figure S10. Validation of SynthEx with TCGA head and neck squamous cellular carcinoma SNP and whole exome data. Figure S11. Statistics of SynthEx with TCGA head and neck squamous cellular carcinoma SNP at varying bin sizes and compared to other whole exome methods. (PDF 6411 kb) [file 13059_2017_1193_MOESM2_ESM.pdf]

Figure S1.

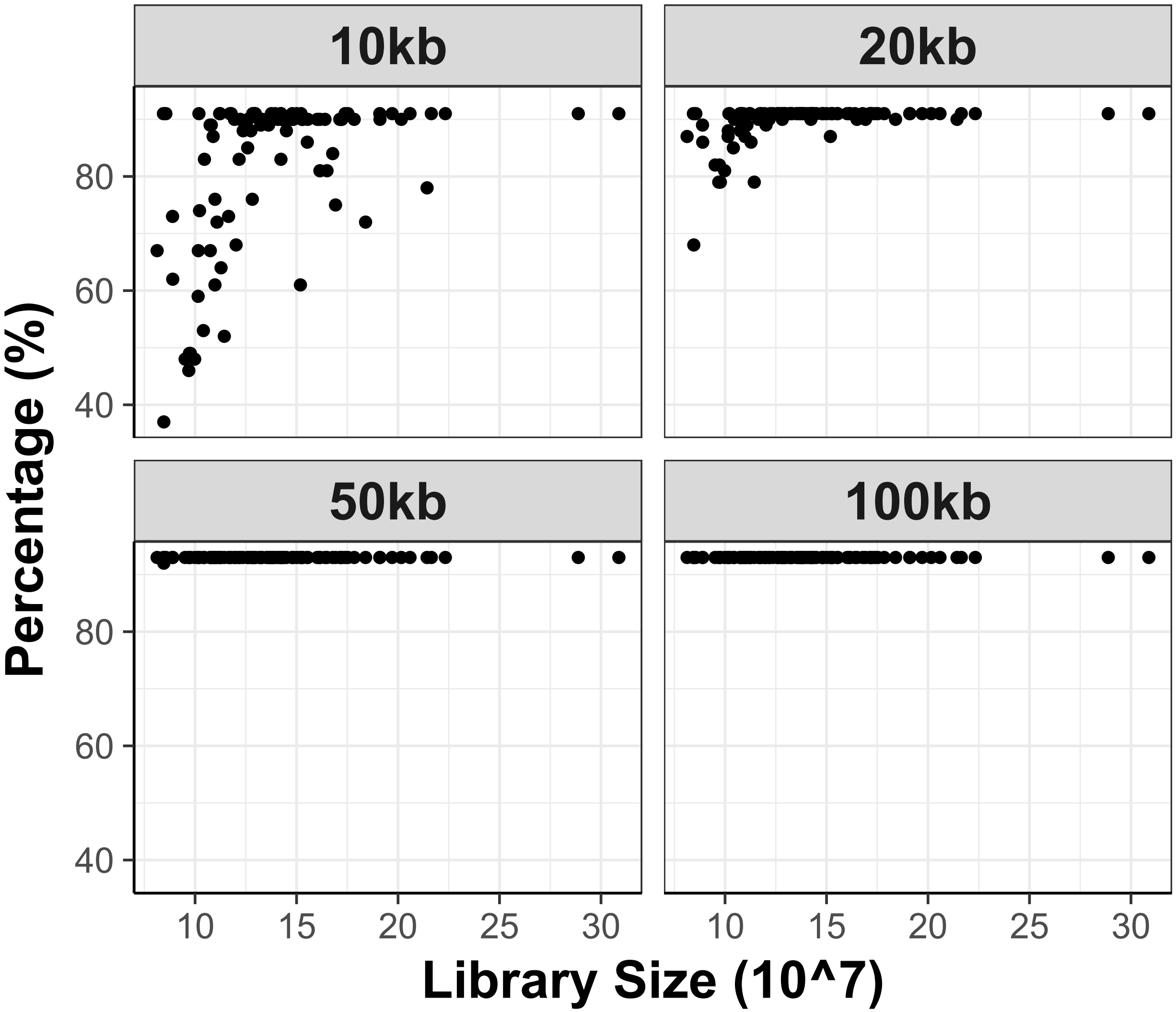

Figure S1. Coverage of bins at varying window sizes.
Non-overlapping bins were tested for a 10 kb, b 20 kb, c 50 kb, and d 100 kb bin sizes. Total percent of bins with at least 50 reads were plotted for library sizes from 8-30 million reads.

Figure S2.

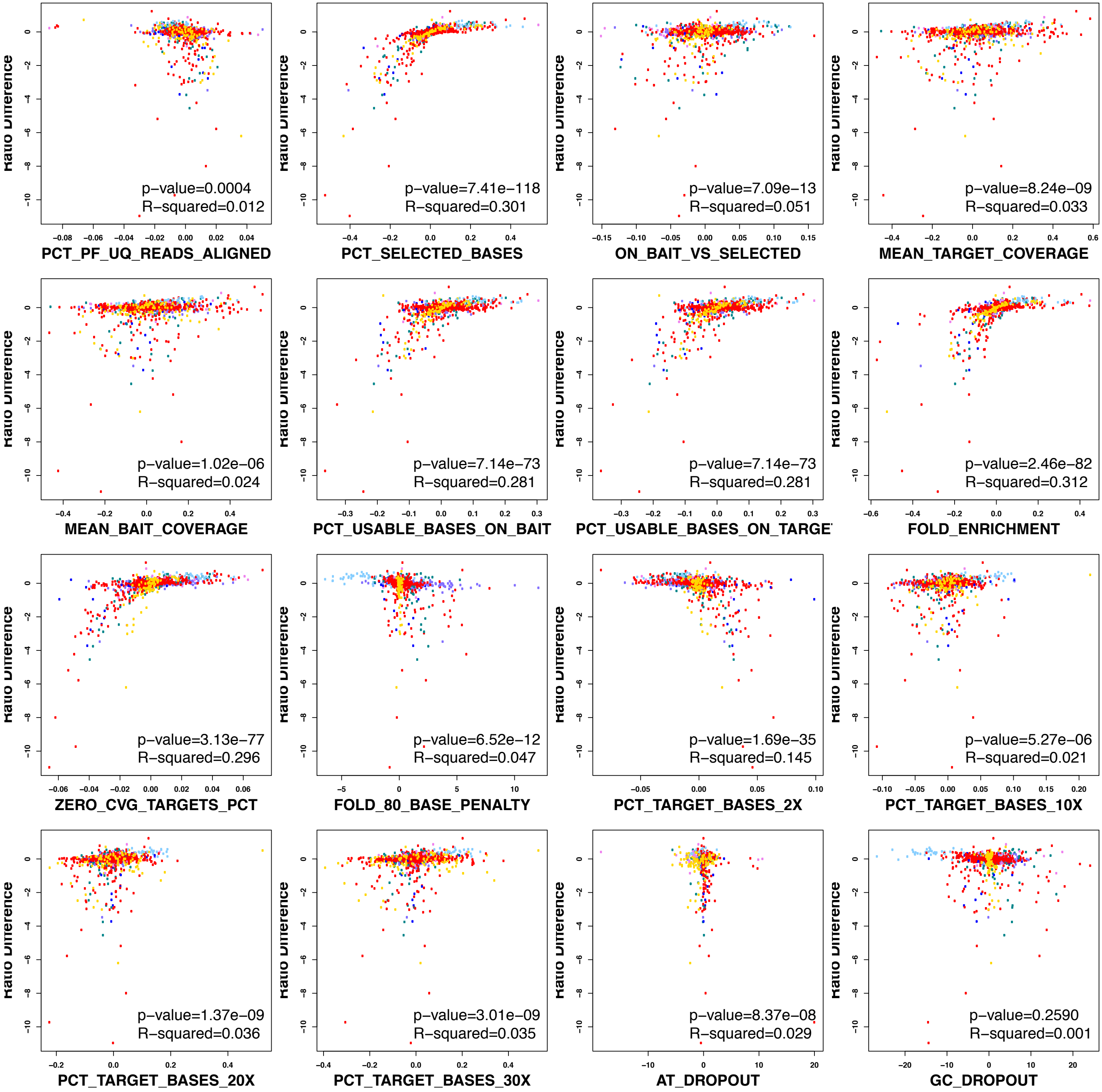

Figure S2. Visualization of sequencing quality metrics. Ratio differences (RD) of the matched tumor – normal pair were plotted against each of the Picard metric differences for matched tumor – normal pair. Linear model p value and coefficients of determination (R^2) are reported. Points are colored based on group membership identification in Figure S4.

Figure S3

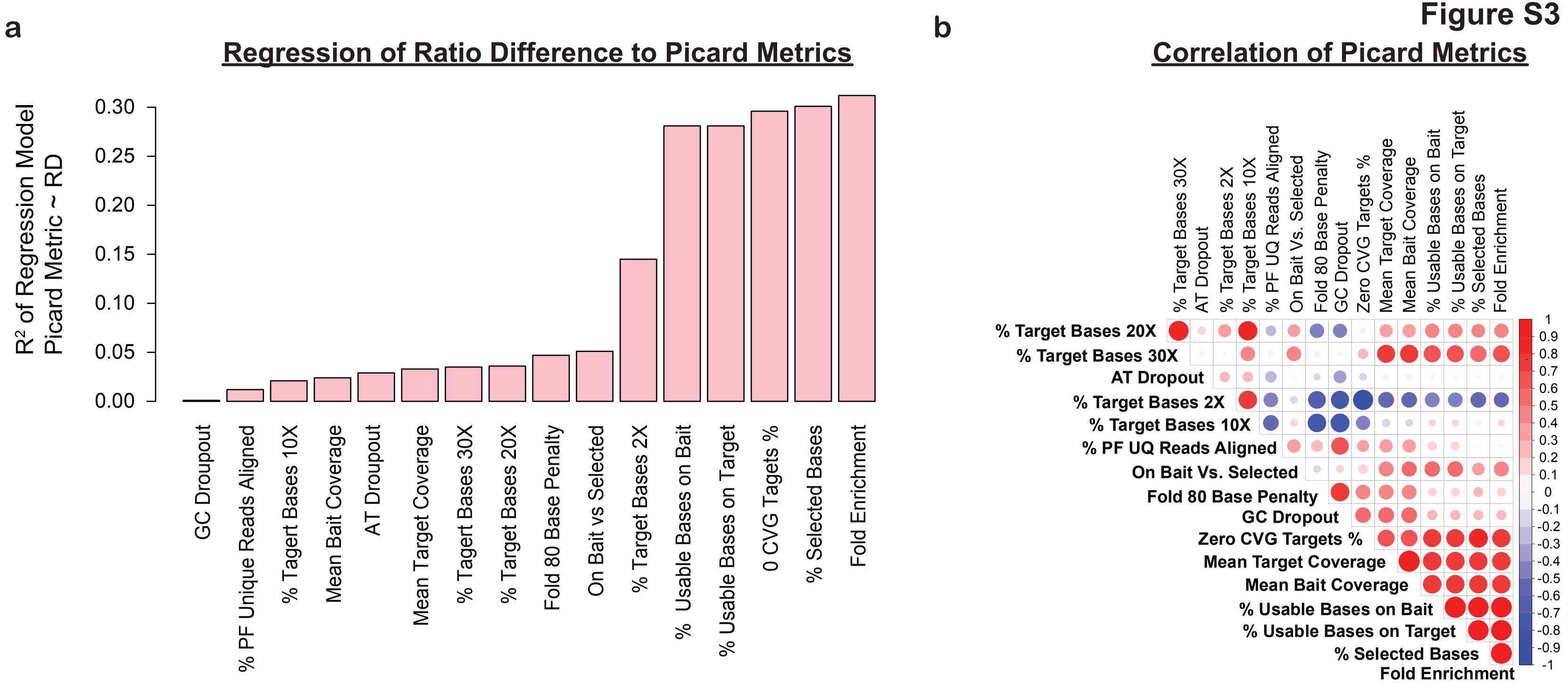

Figure S3. Condensed summary of the influence of Picard metrics on ratio differences (RD) and correlation within the Picard metrics. a Barplot demonstrating the coefficients of determination (R^2) for each Picard metric from the linear model comparing the dependence of RD on the difference in Picard metrics between matched tumor and normal from the TCGA 989 breast cohort. **b** Correlation coefficients for quality metrics compared to all other quality metrics.

Figure S4.

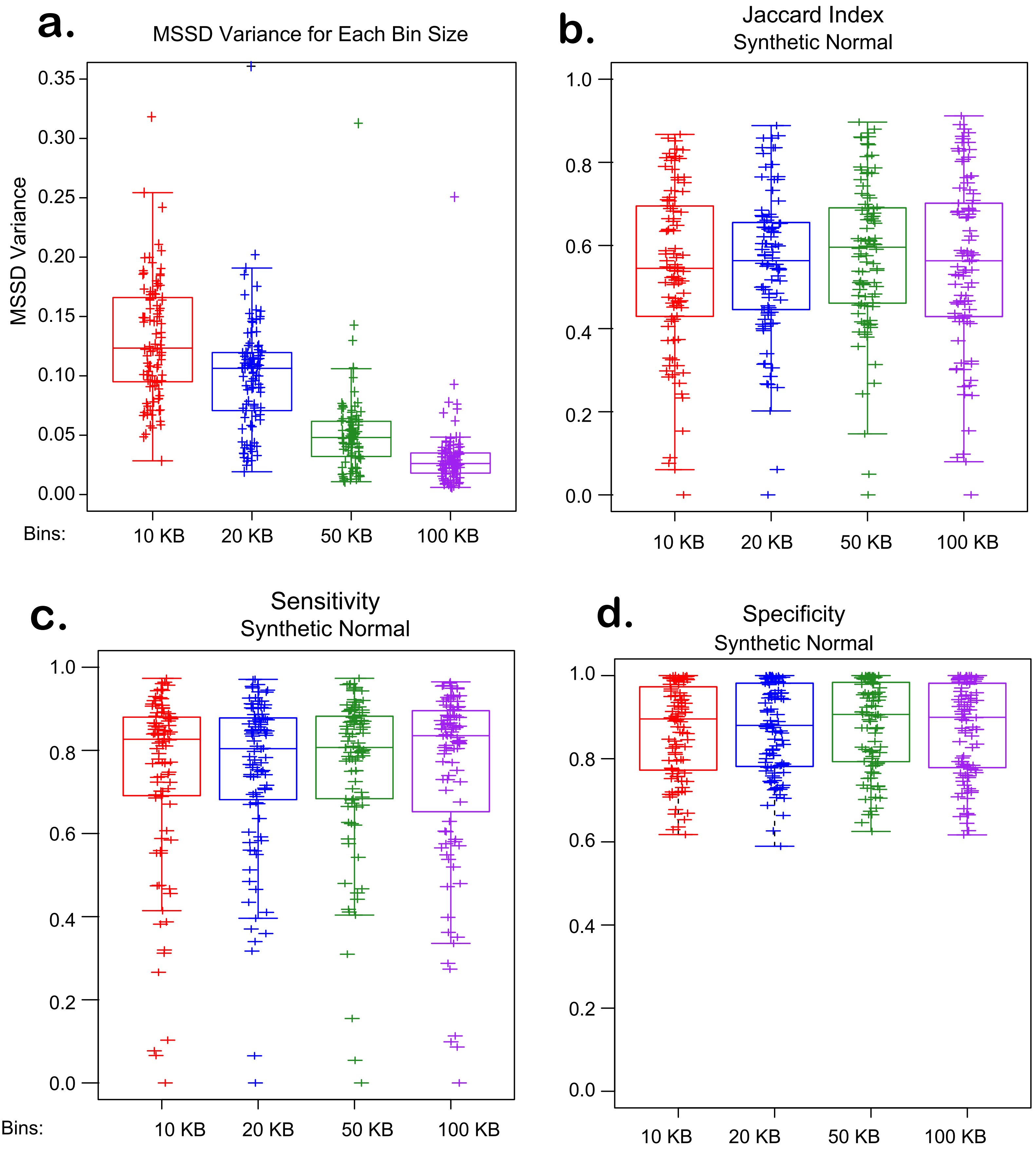

Figure S4. Statistical comparison of varying bin sizes for SynthEx.
SynthEx from synthetic normals based on fold change and libary size at 10, 20, 50, and 100 KB non-overlapping bin sizes were statistically compared to Array SNP data as the gold standard: **a.** mean successive square difference **b.** Jaccard Index, **c.** Sensitivity, and **d.** Specificity

Figure S5

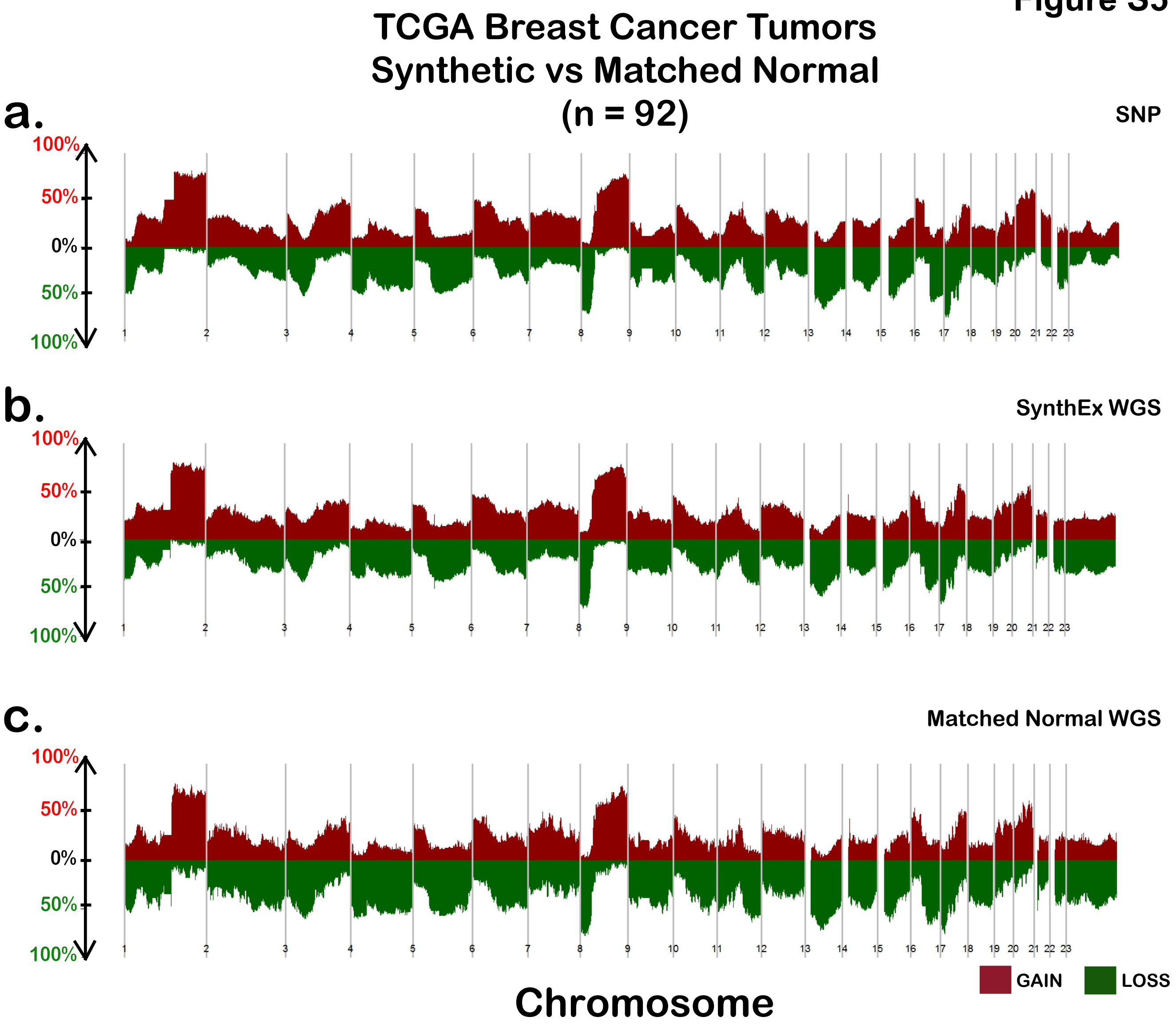

Figure S5. Differences in CNA landscape compared to whole genome with and without a synthetic normal. CNA genomic landscape frequency plots from SWITCHplus using **a** SNP arrays, **b** WGS using SynthEx, and **c** WGS using only matched pairs. Segments of copy number gains are plotted above the x-axis in red and segments of copy number loss are plotted below the x-axis in green. The frequency of an alteration within the 98 breast cancers in each tool is indicated on the y-axis from 0-100%.

Figure S6

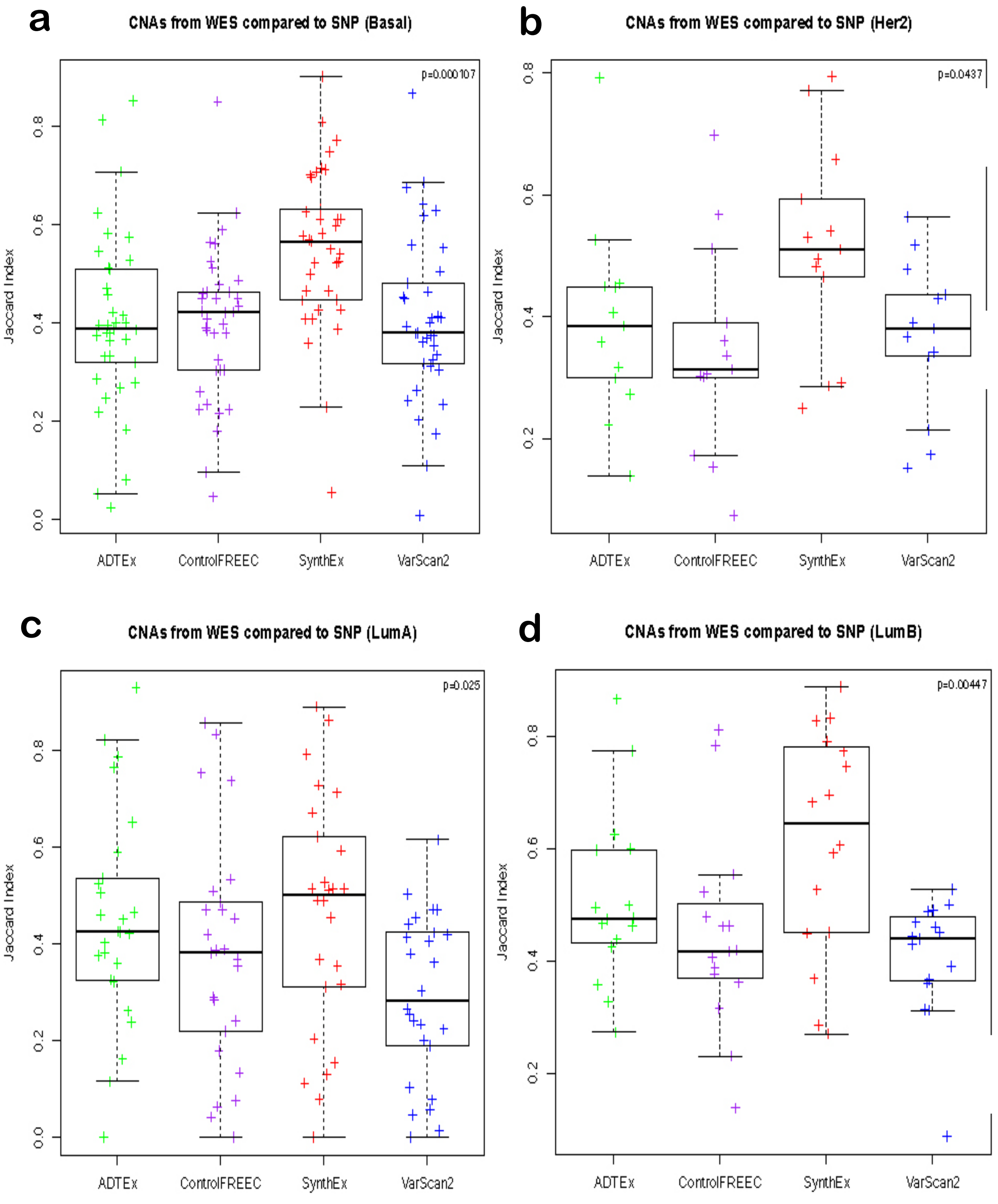

Figure S6. Jaccard Index of WES tools for each breast cancer subtype. Box-and-whisker plots of individual tool-based jaccard index values comparing CNAs from WES tools ADTEx, control-FREEC, SynthEx, and VarScan2 to CNAs from SNP arrays separated by breast cancer molecular subtype: **a** Basal-like, **b** Her2-enriched, **c** Luminal A, and **d** Luminal B breast cancers. ANOVA p values are reported.

Figure S7

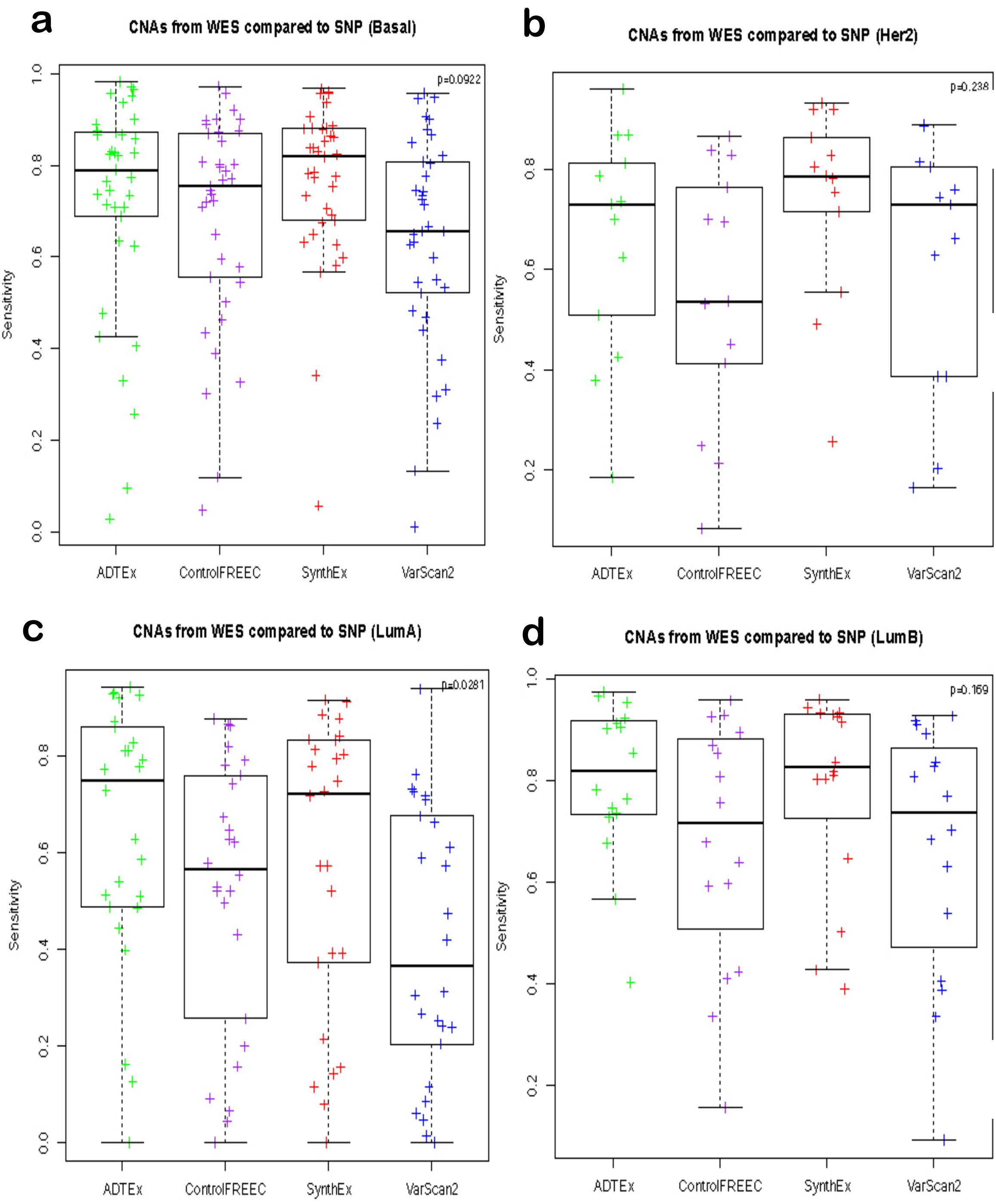

Figure S7. Sensitivity of WES tools for each breast cancer subtype. Box-and-whisker plots of individual tool-based sensitivity comparing CNAs from WES tools ADTEx, control-FREEC, SynthEx, and VarScan2 to CNAs from SNP arrays separated by breast cancer molecular subtype: **a** Basal-like, **b** Her2-enriched, **c** Luminal A, and **d** Luminal B breast cancers. ANOVA p values are reported.

Figure S8

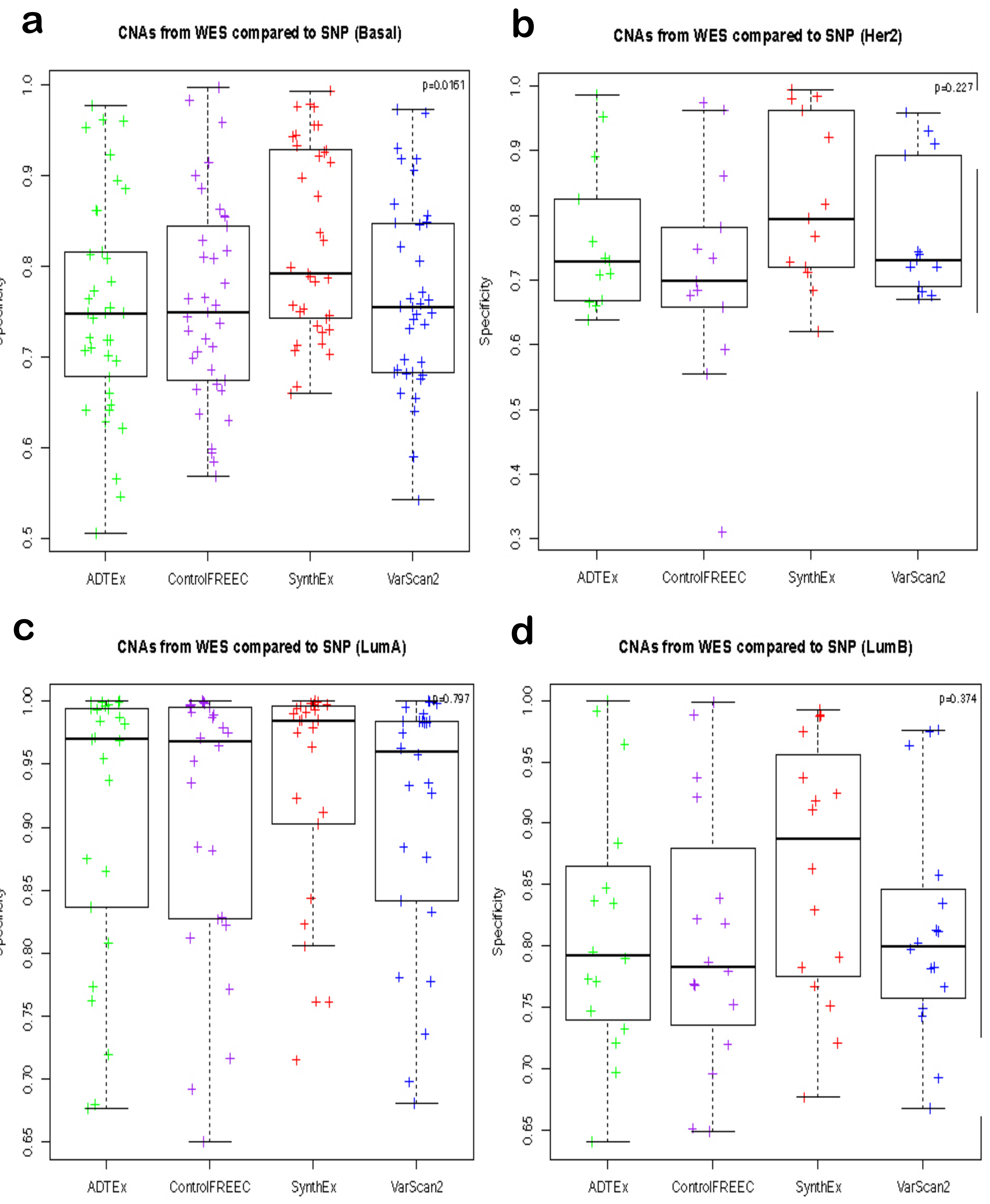

Figure S8. Specificity of WES tools for each breast cancer subtype. Box-and-whisker plots of individual tool-based specificity comparing CNAs from WES tools ADTEx, control-FREEC, SynthEx, and VarScan2 to CNAs from SNP arrays separated by breast cancer molecular subtype:
a Basal-like, **b** Her2-enriched, **c** Luminal A, and **d** Luminal B breast cancers. ANOVA p values are reported.

Figure S9

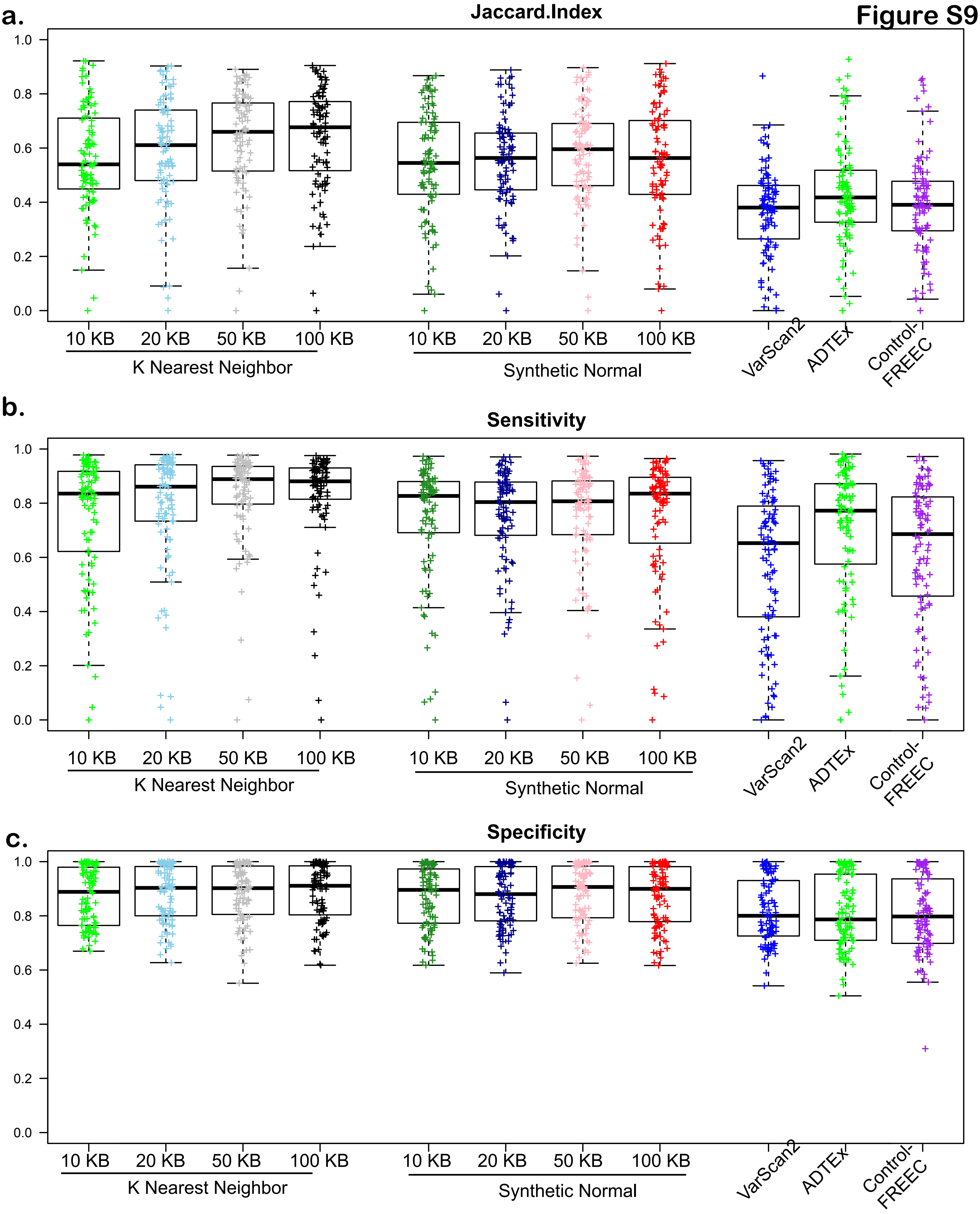

Figure S9. Comparing all SynthEx strategies to other CN detection methods with TCGA BRCA. Varying SynthEx non-overlapping bin sizes from 10 - 100 KB and compared to VarScan2, ADTEx, and Control-FREEC using SNP Array as the gold standard for a. Jaccard Index, b. sensitivity and c. specificity.

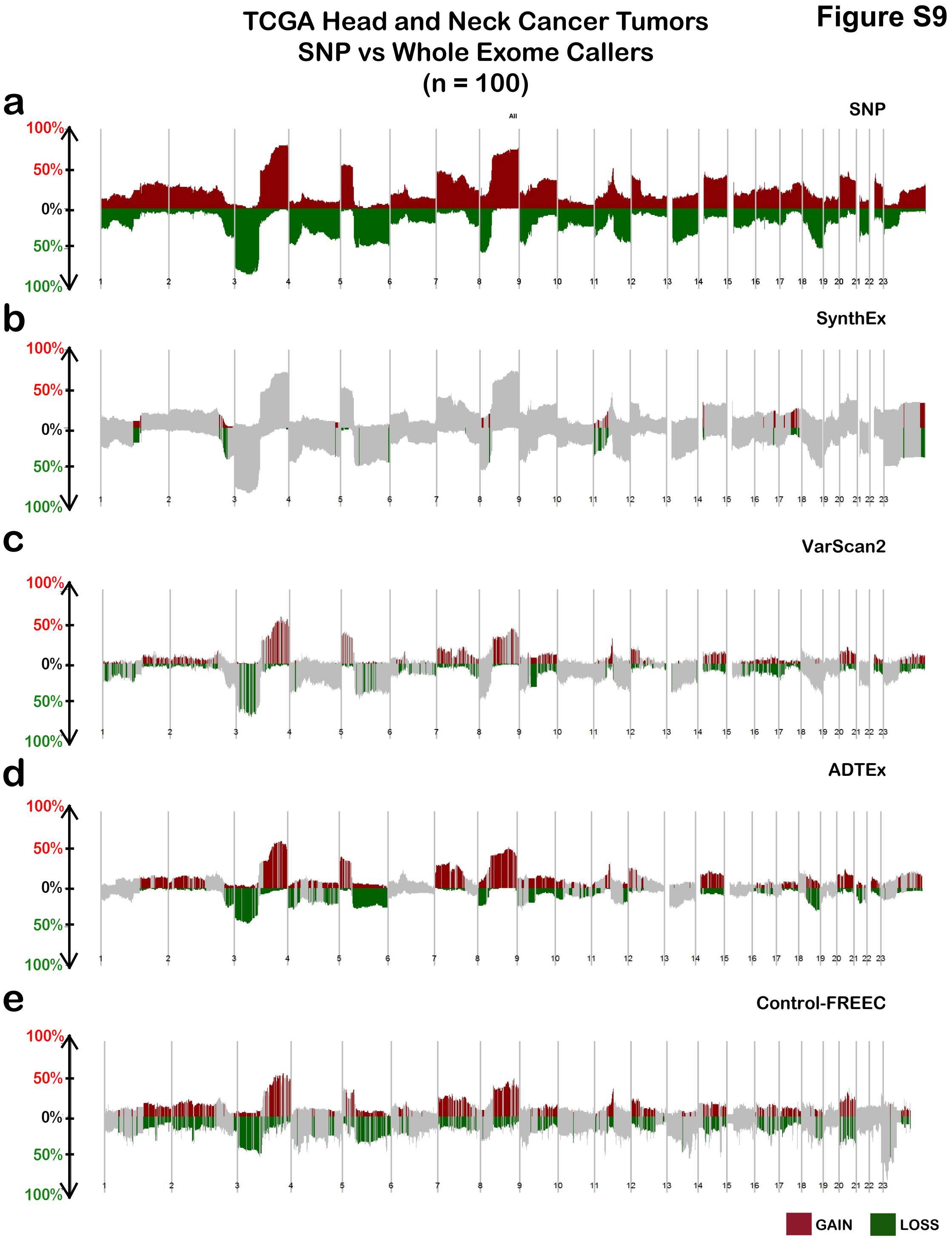

Figure S9. Validation of SynthEx with TCGA head and neck squamous cellular carcinoma SNP and whole exome data. HNSC CNAs genomic landscape frequency plots from SWITCHplus using **a** SNP arrays, **b** SynthEx, **c** VarScan2, **d** ADTEx, and **e** control-FREEC. Segments of copy number gains are plotted above the x-axis in red and segments of copy number loss are plotted below the x-axis in green. The frequency of an alteration in each tool is indicated on the y-axis from 0-100%.

Figure S10

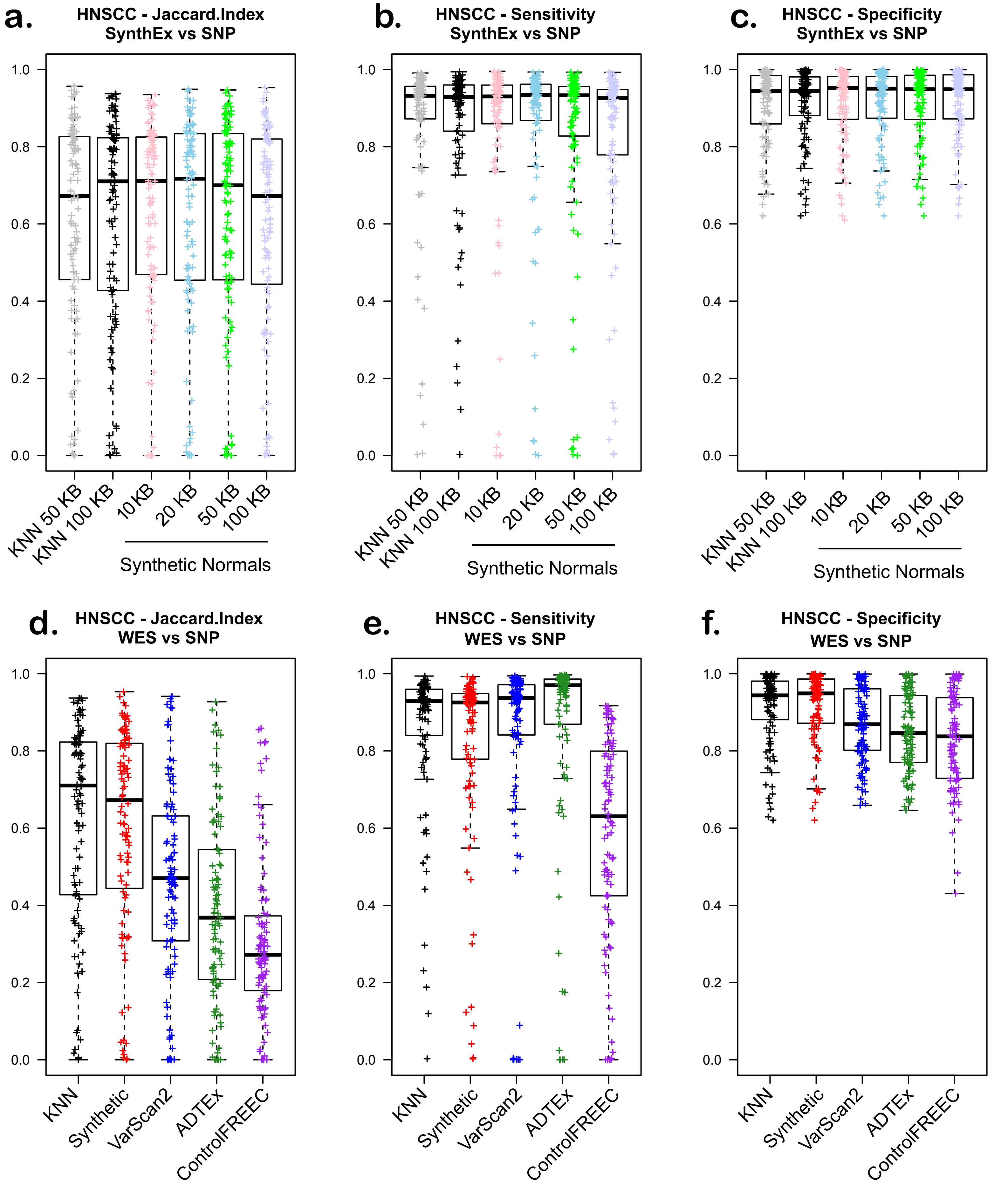

Figure S10. Validation of SynthEx Synthetic Normals on the TCGA Head and Neck Squamous Cell Carcinoma Dataset. Comparison of varying non-overlapping bin sizes for SynthEx KNN (grey/black) and Synthetic Normals to Array SNP data for a. Jaccard Index, b. sensitivity, and c. specificity. Comparing 100 kB non-overlapping bin sizes usinig SynthEx KNN and Synthetic Normals to VarScan2, ADTEx, and ControlFREEC for d. jaccard index, e. sensitivity, and f.specificity.
